# Influence of Gender and SNPs in *GPX1* Gene on Biomarkers of Selenium Status in Healthy Brazilians

**DOI:** 10.3390/nu8050081

**Published:** 2016-05-05

**Authors:** Janaina L. S. Donadio, Elvira M. Guerra-Shinohara, Marcelo M. Rogero, Silvia M. F. Cozzolino

**Affiliations:** 1 Department of Food and Experimental Nutrition, Faculty of Pharmaceutical Sciences, University of São Paulo, São Paulo 05508-900, Brazil; smfcozzo@usp.br; 2 Department of Clinical and Toxicological Analyses, Faculty of Pharmaceutical Sciences, University of São Paulo, São Paulo 05508-900, Brazil; emguerra@usp.br; 3 Department of Nutrition, Faculty of Public Health, University of São Paulo, São Paulo 01246-904, Brazil; mmrogero@usp.br

**Keywords:** selenium (Se), single nucleotide polymorphisms (SNPs), biomarkers, glutathione peroxidase (GPx)

## Abstract

Selenium (Se) status varies worldwide as a result of natural variation of Se content in soils, dietary pattern, and the presence of SNPs. Further, Se status in Brazilians and its relationship between genetic variation and Se biomarkers is unknown. This work investigated the association between SNPs in glutathione peroxidase genes and biomarkers of Se status in healthy Brazilians. The study was conducted in 116 healthy adults in São Paulo, Brazil. Plasma and erythrocyte Se were measured by HGFAAS. Erythrocyte GPx (eGPx) activity was measured spectrometrically in a biochemical analyzer. Genotypes were determined by real-time PCR using Taqman^®^ Assays. eGPx activity was higher in females compared with males. Lower erythrocyte Se concentrations were found in heterozygous GC carriers for *GPX1* rs8179169. eGPx activity was higher in females with the common genotypes, except for rs8179169. GC carriers for rs8179169 had lower erythrocyte Se in both genders, and only male carriers of the variant alleles of both rs1050450 and rs1800668 had higher eGPx activity. In conclusion, the genotype for SNPs in *GPX1* and gender affected biomarkers of Se status in this pilot study with healthy Brazilians.

## 1. Introduction

Selenium (Se) is a fundamental micronutrient for human nutrition. As the amino acid selenocysteine (Sec), Se is incorporated into the active site of selenoproteins coded by 25 genes that have a wide range of functions in human physiology [1,2]. A major function of Se is its role in the defense against oxidative stress as component of the glutathione peroxidase (GPx) enzymes. Other selenoproteins with oxidoreductase function are the Tioredoxin Reductase family of enzymes. These enzymes are part of the major dissulfide cellular redox system [3,4]. Selenoprotein P (SePP) is other important selenoenzyme which may have an antioxidant function, besides its major function as a Se transporter to the tissues [3]. It has been demonstrated, *in vitro*, that SePP can reduce phospholipid hydroperoxides [5] and protect plasma Low density lipoprotein LDL from oxidation [6]. Apart from its antioxidant function, Se is also related to immune function, inflammation [7,8], reproduction and male fertility, brain function, thyroid function [2,9], cardiovascular disease [10], diabetes and cancer [2].

Se nutritional status varies worldwide because the Se content in food is related to the amount in the soil, which depends on both the mineral composition of rock and its bioavailability [11]. Thus, the plasma Se concentrations are variable in different populations around the world. For instance, plasma Se is higher in the USA compared to the South islands of New Zealand [12].

At present, the most regularly used biomarkers of Se status are plasma Se, plasma GPx activity, and plasma SePP [13], all of which are related to Se intake, especially plasma Se. This biomarker can be used to predict Se intake in non-deficient populations consuming Se mostly as selenomethionine (SeMet), as the Se intake cannot be calculated correctly because food composition tables do not reflect the variation of the Se concentration in different soils [14]. In spite of the lack of a reference value, ranges of Se intake and plasma Se levels have been defined in order to improve biological functions, such as maximization of SePP and GPx activities [15]. Plasma Se was the biomarker of choice in several studies because of its sensitivity to a greater range of intake compared with erythrocyte GPx activity (eGPx). The latter is useful only in subjects with low Se status because it reaches a plateau in individuals with higher Se status [13,16]. This sensitivity of plasma Se is explained by the increase of the non-specific compartment in response to Se supplementation, especially in individuals with plasma Se > 70 µg/L [14].

In addition, these biomarkers may also be influenced by factors other than Se intake. In particular, it has been observed that gender, age, smoking status and genetic variations, mostly single nucleotide polymorphisms (SNPs) in selenoprotein genes, affect the biomarker levels in response to Se supplementation [17,18]. The *GPX1* rs1050450 SNP, a C > T substitution that changes proline to leucine in position 198 of the protein, has been associated with lower eGPx activity [19], lower plasma Se levels and a lower correlation of plasma Se and eGPx activity [20]. In addition, another SNP was discovered in the *GPX4* gene, rs713041, which is a C>T substitution located in the 3’UTR, the region of the mRNA that is essential for Sec insertion. This SNP was related to leukotriene metabolism [21]. Furthermore, both cell culture and human studies have supported the hypothesis that this SNP has functional significance [21,22]. Overall, despite the lack of consistent results, the work performed to date suggests that these SNPs in *GPX1* and *GPX4* genes may be associated with chronic disease risk and biomarkers levels. At present, there is no information available regarding the Se status in the general Brazilian population or the relationship between genetic variation in selenoprotein genes and the concentrations of biomarkers of Se status. Therefore, this study was conducted to investigate the influence of SNPs in glutathione peroxidase genes on biomarkers of Se status in healthy Brazilians. Here, we report that a SNP in *GPX1* gene is associated with lower erythrocyte Se and higher eGPx activity, which indicates that this SNP might be functional.

## 2. Methods

### 2.1. Study Population

A total of 116 unrelated healthy volunteers aged 20 to 50 years (males and females) were recruited by poster advertisement in Sao Paulo University, Sao Paulo, Brazil, in 2010. The inclusion criteria were no intake of vitamin and mineral supplements, no diagnosis of cancer, diabetes or cardiovascular disease, no excessive alcohol consumption and no intake of anti-inflammatory drugs. Volunteers completed a lifestyle questionnaire and a 3-day dietary food record. They were contacted by electronic mail or telephone to schedule the blood sampling in the following week. For all participants, their height and weight were measured, and the body mass index (BMI) was calculated. The study protocol was conducted in accordance with the Declaration of Helsinki and was approved by the Ethics Committee of the Faculty of Pharmaceutical Sciences (CAAE: 0023.0.018.000-09). Written informed consent was obtained from all of the subjects before blood sampling.

### 2.2. Blood Collection and Processing

Fasting blood samples (15 mL) were drawn by venipuncture into three 5 mL EDTA tubes. An aliquot of 1.5 mL of EDTA whole blood was used for DNA extraction and subsequent genotyping. Plasma was separated by centrifugation at 1147 *rcf* for 15 min at 4 °C. The erythrocyte pellet was washed three times with 5 mL sterile 9 g/L NaCl solution, slowly mixed by inversion, and centrifuged at 12,745 *rcf* for 10 min (C5408, Eppendorf, Hamburg, Germany) at 4 °C, and the supernatant fluid was discarded. Aliquots of whole blood, plasma and erythrocytes were frozen at −80 °C in sterile, demineralized tubes until the experiments were performed.

### 2.3. Blood Se Concentrations and Erythrocyte GPx Activity

Erythrocyte and plasma Se levels were analyzed by hydride generation flame atomic absorption spectrometry [23]. Samples were prepared by digestion with 68% nitric acid (Merck, Darmstadt, Germany) and heating at 150 °C. After volatilization of the organic material, solutions were reduced from Se^VI^ to Se^IV^ by the addition of 5 mL 1.2 N hydrochloric acid and heating at 100 °C for 2 h. Then, samples were diluted to 25 mL in ultrapure water, and a calibration curve was created with concentrations of 0, 0.1, 0.3, 0.5 1.0, 3.0, and 5.0 mg/L. The method was validated using the lyophilized human reference control Seronorm Trace Elements Serum and Whole Blood (Sero AS, Billingstad, Norway), and reproducibility was achieved by sample analysis in triplicate (technical replicates).

Erythrocyte GPx activity (eGPx) was assessed using the Ransel kit (Ransel 505; RANDOX Laboratories, Crumlin, UK) according to the manufacturer’s instructions. Briefly, aliquots of 50 µL erythrocytes were mixed into 1 mL diluting solution, followed by 5 min of incubation and the addition of 1 mL 2× Drabkin’s reagent. Enzyme activity was evaluated spectrophotometrically at 37 °C at a wavelength of 340 nm using a biochemical analyzer (Labmax 240, Labtest, Minas Gerais, Brazil). The hemoglobin (Hb) concentration was also determined to express eGPx activity in U/g Hb.

### 2.4. SNP Selection, DNA Extraction and Genotyping

The SNPs analyzed were in *GPX1-4* genes, and those in the coding region were all non-synonymous SNPs (nsSNPs) and were expected to alter the amino acid in the protein sequence. This selection was performed using dbSNP database from National Center for Biotechnology Information (NCBI) [24] and the online software PolyPhen, which is designed to predict functional effects of nsSNPs [25]. All of the nsSNPs selected in dbSNP were submitted to Polyphen, and those that the software indicated as potentially affecting protein function were included in the study. The SNPs selected by this method were rs1050450, rs8179169, and rs4991448 for *GPX1*; rs17881652 and rs17880492 for *GPX2*; rs8177445 for *GPX3*. Moreover, the rs3811699 and rs1800668 in the *GPX1* gene were considered because they are likely in the same haplotype as rs1050450 in a Japanese population [19]. Another SNP in the *GPX4* gene was also included; although it occurs in the gene region corresponding to the 3’UTR, it is important for Sec insertion [18]. Four SNPs in the *GPX1*, *GPX2* and *GPX3* genes were excluded (rs4991448, rs17881652, rs17880492, rs8177445) because there were only wild-type genotypes after the first screening. Therefore, only four SNPs in *GPX1* (rs1050450, rs8179169, rs3811699 and rs1800668) and one SNP in *GPX4* (rs713041) were analyzed.

Total genomic DNA was extracted from frozen whole blood samples using GE Illustra Blood GenomicPrep Mini Spin Kit (GE Healthcare, Waukesha, WI, USA) and the final concentration was measured using the Nanodrop ND 1000 spectrophotometer (Thermo Scientific, Waltham, MA, USA). Genotyping was performed using endpoint real-time PCR with a TaqMan SNP Genotyping Assay from Applied Biosystems (Life Technologies, Thermo Scientific, Waltham, MA, USA). The reactions were run on the StepOne Real-Time PCR System (Life Technologies, Thermo Scientific, Waltham, MA, USA). Genotypes were determined by performing an endpoint read after amplification.

### 2.5. Statistical Analysis

The results were first presented as the geometric mean and 95% CI (confidence interval) for continuous variables. Categorical variables were expressed as number and percentage. Allele frequencies were estimated using the gene counting method, and Hardy-Weinberg equilibrium was determined for all genotypes using the χ^2^ test. The differences on biomarkers concentrations were analyzed by Mann-Whitney or Kruskall-Wallis tests. For all the statistical analysis, variant homozygous and heterozygous genotypes were considered together and compared with the wild type, except for rs713041 in *GPX1*, which had a higher variant allele frequency. Linear regression was used to investigate how the biomarkers were correlated. Differences were considered significant at *p* < 0.05. The analyses were performed using the Statistical Package for the Social Sciences software version 17.0 for Windows (SPSS, Chicago, IL, USA) and GraphPad Prism (GraphPad Prism version 5.00 for Windows, GraphPad Software, San Diego, CA, USA).

## 3. Results

### 3.1. Demographic Data

Table 1 shows demographic and biochemical characteristics of the volunteers. These data are separated by gender. The mean age was 28.1 years, and 62.1% of the subjects had a BMI within the normal range. Fewer than 10% of the group were smokers, only 65% reported alcohol consumption, and 64% were physically active. A family history of non-communicable chronic diseases (NCCD) was reported by 79% of the volunteers.

In relation to the biomarkers of Se status, the mean plasma Se and erythrocyte Se was 53.2 µg/L and 53.3 µg/L, respectively, for the total group. When these data were stratified by gender, no differences were observed for plasma and erythrocyte Se. However, there was a significant difference in eGPx activity, which was higher in females than in males (*p* < 0.001). It should be noted that this group of healthy adults is considered sub-optimal in Se status based on these blood levels.

**Table 1:** Demographic and biochemical characteristics of the study volunteers.

	Total (*n* = 116)	Males (*n* = 44)	Females (*n* = 72)	*p* Value *
Age (years)	28.1 (27.0–29.2)	29.7 (27.5–31.8)	27.6 (26.2–29.0)	0.139
BMI				
<18.5	8 (6.9)	0	8 (11.1)	**<0.001**
18.5–25	72 (62.1)	21 (47.7)	51 (70.8)	
>25	36 (31)	23 (52.3)	13 (18.1)	
Smoking	7 (7.1)	2 (5.6)	5 (8.1)	1.000
Alcohol consumption	64 (65.3)	27 (75.0)	37 (59.7)	0.124
Physical activity	63 (64.3)	23 (63.9)	40 (64.5)	0.950
NCCD historical	78 (79.6)	26 (72.2)	52 (83.9)	0.168
Plasma Se (µg/L)	53.2 (49.3–57.2)	53.9 (47.0–60.7)	52.8 (47.8–57.8)	0.785
Erythrocyte Se (µg/L)	53.3 (46.4–60.2)	57.2 (43.5–71.0)	50.9 (43.3–58.4)	0.573
eGPx activity (U/g Hb)	39.8 (36.5–43.1)	34.5 (28.9–40.1)	43.0 (39.0–47.0)	**<0.001**

The data are the geometric mean and CI 95% in parentheses for the following variables: age, BMI, Se intake, plasma Se, erythrocyte Se and eGPx activity. For other variables, the data are number of subjects and percentage in parentheses. The comparison between genders was made using the Mann-Whitney test (age, Se intake), likelihood ratio test (BMI), Chi-square (monthly income, alcohol consumption, physical activity, NCCD historical) and Fisher’s Exact test (smoking). BMI: body mass index; NCCD: non-communicable chronic diseases (cancer, diabetes, obesity, cardiovascular disease); eGPx: erythrocyte glutathione peroxidase activity. Significant *p* values are in bold. * differences between genders.

### 3.2. Genotype Frequencies

Genotype and allele frequencies for *GPX1* and *GPX4* SNPs are shown in Table 2. No variant genotypes were found for *GPX1* SNPs rs8179169 and rs3811699. The variant TT genotype frequency for rs1050450, rs1800668 and *GPX4* rs713041 was 3.4%, 4.3% and 16.2%, respectively. All SNPs were in Hardy-Weinberg equilibrium (*p* < 0.05), except rs3811699.

**Table 2:** Genotypes and allele frequencies for polymorphisms in *GPX1* and *GPX4* genes.

SNPs	Genotypes/Alleles	*n*	%
*GPX1_*rs1050450	CC	56	47.9
	CT	56	47.9
	TT	4	3.4
	C		0.72
	T		0.27
*GPX1*_rs8179169	GG	34	29.1
	GC	82	70.1
	CC	0	0
	G		0.64
	C		0.35
*GPX1*_rs3811699	GG	56	47.9
	GA	60	51.3
	AA	0	0
	G		0.74
	A		0.26
*GPX1_*rs1800668	CC	57	48.7
	CT	54	46.2
	TT	5	4.3
	C		0.72
	T		0.27
*GPX4*_rs713041	CC	45	38.5
	CT	53	44.4
	TT	19	16.2
	C		0.61
	T		0.39

*GPX1*: Glutathione Peroxidase 1 gene; *GPX4*: Glutathione Peroxidase 4 gene.

### 3.3. Effect of Genotypes and Gender on Biomarkers of Se Status

To assess the effect of genotypes on biomarkers of Se status, the data for the total group were stratified by genetic profile (Table 3). No differences in either the plasma Se levels or eGPx activity were observed in different genotypes for the five SNPs studied. However, there was a significant difference in erythrocyte Se levels for SNP rs8179169, where lower values were found in carriers of the GC genotype (*p* < 0.001). The other SNPs had no effects on erythrocyte Se concentrations. No gender effect was observed for either plasma Se or erythrocyte Se. On the other hand, gender had an effect on eGPx activity for all SNPs, with higher activity in females compared to males. This higher eGPx activity was found only in females with the common genotype for four SNPs (rs1050450, rs3811699, rs1800668 and rs713041) and for females with the GC genotype for rs8179169. This result is consistent with those shown in Table 1, in which females had higher eGPx activity than males.

Regarding the genotype effect, mean erythrocyte Se concentrations and eGPx activity were significantly different in relation to SNPs in *GPX1*. Plasma Se was not affected by any genotypes. The erythrocyte Se concentrations were lower in individuals with GC genotype compared to CC genotype for *GPX1_*rs8179169 in both genders (Table 3). This is consistent with the results obtained for the total group. Moreover, males with the combined genotypes CT and TT for SNPs rs1050450 and 1800668 had higher eGPx activity than males with CC genotype.

**Table 3:** Biomarkers of Se status according to gender and genotypes for *GPX1* rs1050450, rs3811699, rs1800668, rs8179169 and *GPX4* rs713041 gene polymorphisms.

SNP	Genotypes	*N*	Total	*N*	Males	*N*	Females	*p* Value *
**Plasma Se, µg/L**								
*GPX1*_rs1050450	CC	56	49.3 (45.1–53.9)	23	52.2 (45.6–59.8)	33	47.4 (41.9–53.6)	0.212
	CT + TT	60	49.8 (44.7–55.4)	21	47.6 (38.4–58.9)	38	51.0 (45.1–57.7)	0.561
	*p* value		0.588		0.655		0.275	
*GPX1_*rs3811699	GG	56	50.5 (46.3–55.1)	22	53.1 (46.3–60.9)	34	48.9 (43.5–54.9)	0.279
	GA	60	48.7 (43.7–54.3)	22	47.0 (38.3–57.6)	38	49.7 (43.7–56.7)	0.668
	*p* value		0.799		0.418		0.718	
*GPX1_*rs1800668	CC	57	50.2 (46.0–54.7)	23	52.2 (45.6–59.8)	34	48.9 (43.5–54.9)	0.384
	CT + TT	59	48.9 (43.9–54.6)	21	47.6 (38.4–58.9)	38	49.7 (43.7–56.7)	0.788
	*p* value		0.967		0.655		0.718	
*GPX1_*rs8179169	GG	34	48.3 (42.5–55.0)	15	54.0 (44.9–64.8)	19	44.3 (36.8–53.3)	0.145
	GC	82	50.1 (46.1–54.4)	29	48.0 (40.8–56.3)	53	51.3 (46.5–56.5)	0.548
	*p* value		0.651		0.360		0.154	
*GPX4_*rs713041	CC	45	52.7 (47.0–59.1)	17	52.8 (41.8–66.6)	28	52.7 (46.1–60.1)	0.953
	CT	52	48.9 (40.3–56.6)	20	47.1 (39.4–56.4)	32	50.0 (44.1–56.6)	0.851
	TT	19	44.4 (36.8–53.6)	7	51.5 (40.1–66.1)	12	40.8 (31.0–53.7)	0.254
	*p* value		0.243		0.655		0.169	
**Erythrocyte Se, µg/L**								
*GPX1*_rs1050450	CC	56	44.3 (38.2–51.4)	23	45.7 (35.9–58.2)	33	43.4 (35.5–52.9)	0.816
	CT + TT	60	44.4 (37.6–52.3)	21	49.1 (37.2–64.9)	38	41.2 (35.0–48.5)	0.598
	*p* value		0.808		0.796		0.892	
*GPX1_*rs3811699	GG	56	45.1 (38.8–52.5)	22	45.8 (35.5–59.0)	34	44.7 (36.7–54.5)	0.993
	GA	60	43.6 (37.1–51.3)	22	48.9 (37.5–63.7)	38	40.8 (33.0–50.5)	0.439
	*p* value		0.847		0.805		0.680	
*GPX1_*rs1800668	CC	57	45.1 (38.9–52.4)	23	45.7 (35.9–58.2)	34	44.7 (36.7–54.5)	0.994
	CT + TT	59	43.6 (36.9–51.5)	21	49.1 (37.2–64.9)	38	40.8 (33.0–50.5)	0.433
	*p* value		0.849		0.796		0.680	
*GPX1_*rs8179169	GG	34	63.8 (54.1–75.3)	15	61.3 (44.6–84.3)	19	65.9 (54.7–79.4)	0.425
	GC	82	38.1 (33.6–43.3)	29	41.3 (33.6–50.8)	53	36.5 ( 30.9–43.0)	0.500
	*p* value		**<0.001**		**0.016**		**<0.001**	
*GPX4_*rs713041	CC	45		17	43.9 (32.1–60.1)	28	38.2 (30.7–47.5)	0.717
	CT	52	40.2 (33.8–47.9)	20	51.4 (39.1–67.6)	32	45.6 (36.3–57.3)	0.792
	TT	19	47.8 (40.3–56.6)	7	44.7 (27.2–73.4)	12	46.1 (30.5–69.8)	0.833
	*p* value		0.235		0.588		0.420	
**eGPx activity, U/g Hb**								
*GPX1*_rs1050450	CC	56	33.9 (29.4–39.0)	23	25.6 (20.7–31.8)	33	41.2 (35.0–48.5)	**0.001**
	CT + TT	60	37.1 (32.7–42.0)	21	35.0 (26.5–46.3)	38	38.2 (33.6–43.5)	0.908
	*p* value		0.395		**0.046**		0.314	
*GPX1_*rs3811699	GG	56	35.4 (30.9–40.7)	22	26.8 (21.8–32.9)	34	42.5 (36.0–50.0)	**0.001**
	GA	60	35.6 (31.3–40.5)	22	33.0 (24.6–44.3)	38	37.1 (32.8–42.1)	0.878
	*p* value		0.965		0.130		0.096	
*GPX1_*rs1800668	CC	57	34.6 (30.0–39.9)	23	25.6 (20.7–31.8)	34	42.5 (36.0–50.0)	**<0.001**
	CT + TT	59	36.4 (32.2–41.1)	21	35.0 (26.5–46.3)	38	37.1 (32.8–42.1)	0.899
	*p* value		0.722		**0.046**		0.096	
*GPX1_*rs8179169	GG	34	38.1 (30.9–46.8)	15	30.2 (20.2–45.2)	19	45.7 ( 37.7–55.3)	0.111
	GC	82	34.5 (31.5–38.2)	29	29.5 (24.5–35.6)	53	37.6 (33.4–42.3)	**0.041**
	*p* value		0.138		0.729		0.080	
*GPX4_*rs713041	CC	45	33.6 (28.6–39.4)	17	25.4 (18.6–34.5)	28	39.8 (33.9–46.7)	**0.017**
	CT	52	37.7 (32.7–43.4)	20	32.6 (24.6–43.2)	32	41.3 (35.3–48.2)	0.176
	TT	19	34.4 (28.2–41.9)	7	33.7 (24.1–47.1)	12	34.8 (26.1–46.3)	0.899
	*p* value		0.494		0.421		0.502	

Values are geometric mean and CI 95%. Differences between genders and genotypes tested by Mann-Whitney test. For *GPX4* rs713041, differences between genotypes tested by Kruskall-Wallis Test. Significant *p* values are in bold. * differences between genders.

### 3.4. Effect of Genotypes and Plasma Se on Biomarkers of Se Status

Table 4 shows the biomarkers stratified by plasma Se tertiles and genotypes. Plasma Se was used here as a marker of Se intake. Erythrocyte Se concentrations were different in relation to *GPX1*_rs8179169 genotype across the three tertiles of plasma Se. As observed by the linear regression, erythrocyte Se was significantly influenced by plasma Se (*R*^2^ = 0.25; β = 0.05; *p* < 0.001, Table S1 and Figure S1). Lower erythrocyte Se concentrations were observed in individuals with GC genotype compared to individuals with CC genotype. No differences were observed for eGPx activity.

**Table 4:** Gene effects on biomarkers of Se status according to tertiles of plasma Se.

SNP	Genotypes	Plasma Se (Tertiles)			*p* Value
		<42.5	42.5–55.4	>55.4	
**Erythrocyte Se, µg/L**	total	36.5 * (30.6–43.0)	39.1 (33.5–45.6)	60.9 (49.1–75.5)	**<0.001**
*GPX1*_rs1050450	CC	36.4 * (28.6–46.3)	39.1 * (33.1–46.3)	62.4 (45.5–85.6)	**0.016**
	CT + TT	36.3 * (27.8–47.2)	39.2 (30.1–50.9)	59.6 (43.4–81–9)	**0.034**
	*p* value	0.736	0.612	0.888	
*GPX1*_rs8179169	GG	51.5 * (42.1–63.0)	57.1 (46.5–69.9)	94.3 (62.7–141.8)	**0.004**
	GC	30.9 * (25.1–38.0)	33.1 * (27.8–39.4)	52.4 (41.2–66.6)	**0.003**
	*p* value	**<0.001**	**<0.001**	**0.017**	
*GPX4*_rs713041	CC	28.9 (21.1–39.6)	34.1 (27.2–42.7)	57.5 (43.1–76.8)	**0.008**
	CT + TT	40.4 (33.0–49.4)	42.7 (34.5–52.7)	63.9 (45.7–89.3)	**0.031**
	*p* value	0.068	0.066	0.735	
**eGPx activity, U/g Hb**	total	30.0 (24.9–36.1)	38.5 (33.1–44.8)	38.5 (33.4–44.5)	0.075
*GPX1*_rs1050450	CC	30.1 (23.3–39.0)	39.2 (30.0–51.2)	33.1 (26.2–42.3)	0.266
	CT + TT	29.8 (22.1–40.1)	37.9 (31.4–45.7)	43.7 (36.8–51–8)	0.153
	*p* value	0.872	0.621	0.121	
*GPX1*_rs8179169	GG	36.2 (22.6–58.1)	35.7 (25.4–50.3)	43.4 (30.6–61.6)	0.065
	GC	27.5 *(22.9–33.0)	39.7 (33.4–47.3)	37.0 (31.4–43.5)	**0.007**
	*p* value	0.061	0.659	0.430	
*GPX4*_rs713041	CC	22.9 * (16.1–32.5)	40.5 (30.8–53.3)	37.0 (29.7–46.1)	**0.013**
	CT + TT	33.9 (27.3–42.2)	37.2 (30.7–45.2)	39.9 (32.4–49.1)	0.653
	*p* value	0.053	0.593	0.602	

Values are geometric mean and CI 95%. Differences between genotypes tested by Mann-Whitney test. Differences between tertiles tested by Kruskall-Wallis test. Significant *p* values are in bold. * different from the highest tertile.

## 4. Discussion

Previous studies have shown that genetic variations in *GPX4* and *SEPP1* genes affect the response to Se supplementation [17,18,26] and the genotype for SNPs in *GPX1* and *GPX4* genes both reportedly affect GPx activity [18,20,27]. The present work extends these earlier observations by demonstrating that the genetic profile, gender and Se intake level affect biomarkers of Se status in healthy Brazilian individuals without a supplementation. Moreover, a new *GPX1* SNP in the coding region changing Arg to Pro in position 5 of the protein (rs8179169) was described and could be functional.

The biomarkers of Se status used in this study were plasma Se, erythrocyte Se and eGPx activity. Plasma Se concentrations (53.2 µg/L) in this study were very low compared with those observed in other healthy populations, such as the USA (142 µg/L) [16], New Zealand (111.6 µg/L) [28] and UK (90.8 µg/L) [18]. As it has been established that Se intake can be predicted by plasma Se concentrations [14,26], this lower concentration could be a consequence of low Se intake and the low Se content in foods in this southern region of Brazil. Considering this plasma Se concentration, plasma GPx and SePP would not be fully expressed, as it has been determined that plasma Se should be of 80 µg/L to maximize selenoprotein expression [14]. Although erythrocyte Se has not been the most used biomarker to assess Se status in recent studies, it had been used in studies in Europe, China and New Zealand, showing a response to selenium supplementation and it is considered a useful long-term marker of Se status [29]. When the present plasma Se values were compared with those proposed by Thomson [15], only 2.6% of the subjects were within the physiological requirement for maximal plasma GPx and SePP activities (78.9–94.7 µg/L). The present results suggest that these healthy Brazilian subjects had marginal inadequate Se status and that a Se supplementation would be beneficial.

Polymorphisms in selenoprotein genes coding for the antioxidant enzymes glutathione peroxidase 1 and 4 have been reported in several healthy populations [20,26,27,28,30,31]. However, the genotype distribution for rs1050450 is ethnicity-dependent: in Japanese, the T allele frequency is 0.05, whereas, in Africans-Americans, the frequency is 0.35 [31]. The Brazilian population is very mixed ethnically, and in our study, the T allele frequency for rs1050450 was 0.27, similar to what was observed in a Swedish population [32]. The T allele frequency found for rs713041 in our study was 0.39, which was comparable to the results of other studies that found T allele frequencies of 0.43, 0.45 and 0.46 [26,28,33]. Particularly, this *GPX4* SNP was not dependent on the ethnic group either in this study or in the others.

Although other studies have evaluated the genotype distribution of rs1050450 in Alzheimer’s patients [34], obese women [35] and even in healthy subjects [36,37] in Brazilian populations, this is the first study that has evaluated other SNPs in glutathione peroxidase genes such as *GPX1* rs8179169, which could have a potential biological consequence. Moreover, we observed a higher frequency of GC heterozygotes for *GPX1* rs8179169 compared with the frequency observed in a German population of 100% GG homozygotes [38]. This higher frequency observed in our Brazilian population could be a result of ethnic differences and the small sample size of the group.

The effect of genotype on biomarkers of Se status was influenced by gender and intake. The *GPX1* rs8179169 SNP, a missense SNP in which Arginine is changed to Proline in position 5 of the protein, had an effect on erythrocyte Se, with lower concentrations in individuals with the GC genotype. This occurred in both genders. Females had higher eGPx activity than males in the same genotypes. The influence of gender on eGPx activity confirms earlier studies that found higher eGPx activity in women, which is likely due to high estrogen levels and hormonal contraceptive use [39]. To our knowledge, the effect of rs8179169 on erythrocyte Se and eGPx activity has not been observed before. The effects of genotype were also observed in eGPx activity, in which males with the variant T allele for rs1050450 and rs1800668 had higher activity than males with the common genotype. This was an unexpected result, as it had been demonstrated that the TT genotype decreases GPx activity [19,40]. Nevertheless, our data included CT and TT genotypes in the same group due to small sample size. Combining those genotypes could have masked the real difference in eGPx activity in one particular genotype. However, it is still not completely established in the literature how this SNP affects eGPx activity since no differences were observed in eGPx1 activity in two other studies [32,41]. One study using lymphocytes cell lines showed that the Leu allele in combination with another polymorphism, a trinucleotyde insertion (GCG) in exon 1, increased GPx1 activity and decreased enzyme thermostability after supplementation with sodium selenite and selenomethionine [42]. A recent study found that Pro allele was more frequent in females with Panic Disorder than in males, which may be related to the development of this mental disorder with a gender specific component [43].

Furthermore, an effect of Se intake and genotypes was observed on biomarkers of Se status. Considering plasma Se as a biomarker of Se intake and stratifying the data into tertiles of plasma Se, the genotype influence was observed only for SNP rs8179169 in erythrocyte Se. We found lower erythrocyte Se in individuals with the GC genotype across all plasma Se tertiles. This suggests that this SNP might be functional. However, further studies are needed to elucidate its role on Se metabolism and regulation of selenoprotein expression and activity. The genotypes for SNPs in *GPX1* gene did not affect Plasma Se, possibly because no SNPs in the selenoprotein P gene (*SEPP1*) were assessed. SePP is the most important Se transporter [44] and some studies have suggested that genetic variation in this gene is associated with changes in plasma Se and SePP concentrations [17].

Although this pilot study had not the objective of associating the genetic variation in selenoprotein genes with risk of diseases, it should be noted that the polymorphisms mentioned here, mainly *GPX1* rs1050450 and *GPX4* rs713041, have been associated with cancer risk. Leu allele for *GPX1* rs1050450 was associated with decrease in eGPx activity and increase of breast cancer risk in a Danish population [40]. Another Danish study showed that Leu allele was associated with risk of non-ductal tumors and the association with one SNP in SEPP1 gene (rs3877899) increased this risk [33]. Leu allele was also associated with increased risk for lung cancer [45], bladder cancer [46,47], prostate cancer [48] and cardiovascular disease [19]. The variant T allele for *GPX4* rs713041 SNP was associated with increased risk of mortality by breast cancer [49] and decreased eGPx activity [33].

One of the limitations of this work was the small sample size, which could have masked the genotype effects on GPx activity and other biomarkers of Se status. Additionally, the analysis of the effects of *GPX4* SNP was limited because only the eGPx activity was analyzed; measuring GPx4 lymphocyte activity and protein concentration would be useful in future Brazilian’s works. Besides, the utilization of other biomarkers, such as plasma SePP and GPx3 activity would help understand the relation between Se intake and genotypes in this sub-optimally nourished Brazilian population.

## 5. Conclusions

In conclusion, this pilot work suggests that the genotype for SNPs in *GPX1* gene could have an effect on biomarkers of Se status in a healthy Brazilian population not taking Se supplements. The average plasma Se concentration found in this study is considered low when compared to other healthy populations around the world. Therefore, it should be considered a fundamental argument for local authorities to enhance Se intake by means of fortification of the most consumed food or stimulating the intake of high-content Se food, such as Brazil nuts. The influence of gender and intake with genotypes potentially has a crucial application in future personalized dietary recommendations. Further work with a bigger sample size evaluating the effect of genotypes after a supplementation with a high-content Se food, such as Brazil nuts, would help to obtain a better knowledge of how genetic variants influence the regulation of selenoprotein expression and biomarkers of Se status.

## Supplementary Materials

The following are available online at http://www.mdpi.com/2072-6643/8/2/81/s1, Table S1: Relation between biochemical variables in healthy subjects by linear regression. Figure S1. Relation between biochemical variables in healthy subjects by linear regression.
