# Supplementary material for: Influence of Gender and SNPs in GPX1 Gene on Biomarkers of Selenium Status in Healthy Brazilians
Source: Nutrients. 2016 May 5;8(5):81. doi: 10.3390/nu8050081 (PMC4882653; doi:10.3390/nu8050081)
Supplement: Supplementary file 1 [file nutrients-08-00081-s001.pdf]

Nutrients **2016**, *8*, 81; doi:10.3390/nu8050081

Influence of Gender and SNPs in *GPX1* Gene on Biomarkers of Se Status in Healthy Brazilians

Janaina L. S. Donadio, Elvira M. Guerra-Shinohara, Marcelo M. Rogero and Silvia M. F. Cozzolino

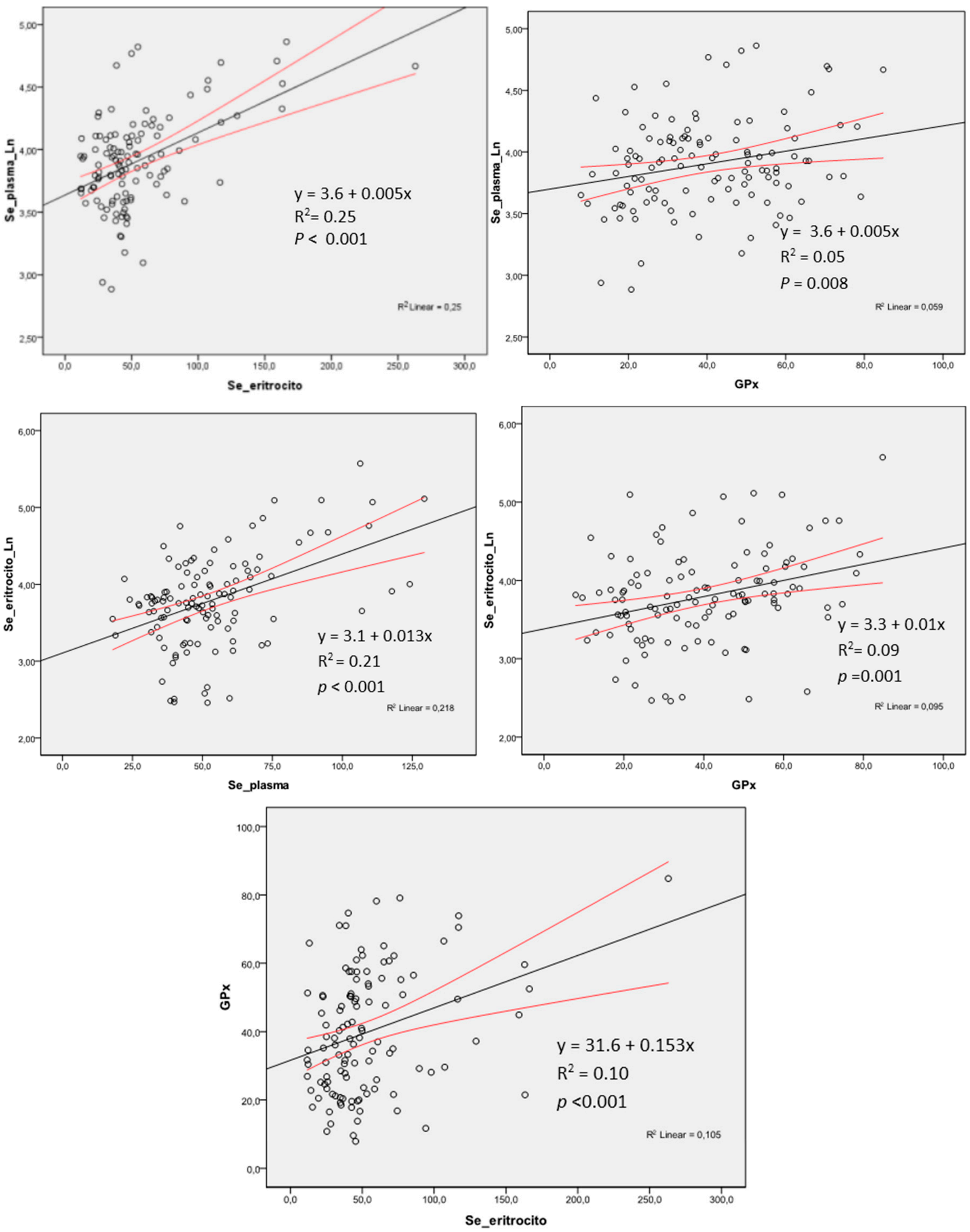

Figure S1. Association between biochemical variables in healthy subjects by linear regression.

Table S1. Relation between biochemical variables in healthy subjects by linear regression.

**Dependent Variables**	**Independent Variables**	**R^2**	**β**	**Standard Error**	***p***
Plasma Se(μg/L) [1]	Erythrocyte Se	0.25	0.005	0.32	<0.001
Erythrocyte Se(μg/L) [1]	Plasma Se	0.21	0.013	0.53	<0.001
GPx(U/g Hb)	Plasma Se	0.05	0.199	17.38	0.009
	Erythrocyte Se	0.10	0.153	16.94	<0.001

[1] Dependent variables used as log transformed.
